# The Vegetable ‘Kale’ Protects against Dextran-Sulfate-Sodium-Induced Acute Inflammation through Moderating the Ratio of Proinflammatory and Anti-Inflammatory LPS-Producing Bacterial Taxa and Augmenting the Gut Barrier in C57BL6 Mice

**DOI:** 10.3390/nu15143222

**Published:** 2023-07-20

**Authors:** Samnhita Raychaudhuri, Md Shahinozzaman, Ujjwol Subedi, Si Fan, Opeyemi Ogedengbe, Diana N. Obanda

**Affiliations:** Department of Nutrition and Food Sciences, University of Maryland, College Park, MD 20742, USA; samnhita@umd.edu (S.R.); mshahin@umd.edu (M.S.); usubedi@umd.edu (U.S.); sfan9@umd.edu (S.F.); ogeds20@umd.edu (O.O.)

**Keywords:** kale, Enterobacteriaceae, proinflammatory lipopolysaccharide, gut barrier, inflammation

## Abstract

Kale (*Brassica oleracea* var. acephala), a food rich in bioactive phytochemicals, prevents diet-induced inflammation and gut dysbiosis. We hypothesized that the phytochemicals protect against the lipopolysaccharide (LPS)-induced acute inflammation which results from gut dysbiosis and loss of gut barrier integrity. We designed this study to test the protective effects of the whole vegetable by feeding C57BL/6J mice a rodent high-fat diet supplemented with or without 4.5% kale (0.12 g per 30 g mouse) for 2 weeks before administering 3% dextran sulfate sodium (DSS) via drinking water. After one week, DSS increased the representation of proinflammatory LPS (P-LPS)-producing genera *Enterobacter* and *Klebsiella* in colon contents, reduced the representation of anti-inflammatory LPS (A-LPS)-producing taxa from *Bacteroidales*, reduced the expression of tight junction proteins, increased serum LPS binding protein, upregulated molecular and histopathological markers of inflammation in the colon and shortened the colons. Mice fed kale for 2 weeks before the DSS regime had a significantly reduced representation of *Enterobacter* and *Klebsiella* and instead had increased *Bacteroidales* and Gram-positive taxa and enhanced expression of tight junction proteins. Downstream positive effects of dietary kale were lack of granuloma in colon samples, no shortening of the colon and prevention of inflammation; the expression of F4/80, TLR4 and cytokines 1L-1b, IL-6, TNF-a and iNOS was not different from that of the control group. We conclude that through reducing the proliferation of P-LPS-producing bacteria and augmenting the integrity of the gut barrier, kale protects against DSS-induced inflammation.

## 1. Introduction

Inflammation is related to several diseases, but Crohn’s disease (CD) and ulcerative colitis (UC), which make up inflammatory bowel disease (IBD), are associated with severe or acute inflammation. In 2017, it was estimated that 6.8 million persons had IBD worldwide, an increase of 85% from 1990, when only 3.7 million had the disease [1]. By 2017, about 3 million of these cases were in the USA and Europe, with incidence and prevalence being much lower in Asia, Africa and South America [1]. The cause of UC is complex and remains unclear; it develops because of a complex interaction of genetic susceptibility, gastrointestinal microbiota, environmental factors and over-reactive immune responses [2]. Because the incidence has been rising in association with industrialization and westernization in less developed countries, it is likely that diet plays a significant role in its pathogenesis and prevention. In this regard, since diet and gut microbiota compositions are closely linked, it is predicted that important clues to the pathogenesis of IBD lie in an analysis of the intestinal microbiota and how it is linked to the pathogenesis of inflammation [2]. Links between IBD and the intestinal microbiota or immune responses include loss of tolerance to intestinal microbiota, impaired tolerance to new microbiota components or an overactive mucosal immune system and the failure of mucosal defense mechanisms to suppress inflammatory stimulus [2,3].

Since the burden of inflammation-related diseases such as IBD and obesity is rising globally, it is important to investigate dietary interventions that are protective by suppressing the inflammatory stimulus and response or preventing gut microbiota dysbiosis. Numerous food-derived bioactive compounds alleviate UC and have been verified in animal models [3]. In this context, cruciferous vegetables (genus *Brassica*) rich in sulfur-containing indolic glucosinolates and aliphatic glucosinolates are associated with better health outcomes and a lower risk of metabolic diseases and other non-communicable diseases. For several years, broccoli and cabbage have been the most widely studied and reviewed cruciferous vegetables. Kale (*Brassica oleracea* var. acephala) is a less recognized member of *Brassica* but has gained popularity and become an important crop in the United States in recent years [4]. Kale ranks high on the list of super foods based on a classification scheme that defines powerhouse fruits and vegetables as foods providing 10% or more of the daily value per 100 kcal of 17 qualifying nutrients [5]. In previous studies, we have shown that dietary supplementation with kale does not attenuate obesity or fat accumulation, but it effectively suppresses the inflammatory response and gut dysbiosis induced by a high-fat (HF) diet. Particularly, it increases the representation of bacterial families associated with health and decreases bacterial families associated with inflammation [6]. Furthermore, it lowers the levels of circulating lipopolysaccharide (LPS) [7], thus indicating a protective effect through changing the gut microbiota function (LPS production) or augmenting gut barrier integrity to prevent LPS entry into the circulation. More recent work has shown that LPS produces differential effects on the immune system, inflammation and gene expression depending on its origin, structure and concentration. Differences in structure arise from the phosphorylation and acylation levels of the lipid A moiety and the structure of the O-antigen polysaccharide [8,9,10]. Gut bacteria from phylum *Proteobacteria* produce LPS that induces TLR4 signaling, and thus inflammation (termed P-LPS), while many species from order *Bacteroidales* produce LPS that does not activate TLR4 (termed A-LPS) and is immunoinhibitory or antagonistic to the effects of LPS from *Proteobacteria* [8,9,10]. Differences in the ratio of these two LPS types are based on the gut microbiota composition, and they determine the impact of the inflammation phenotype that precipitates [8,9].

Herein, we induce dysbiosis and acute inflammation in C57BL/6J mice using dextran sodium sulfate (DSS). While various murine models of intestinal inflammation have been developed to elucidate the pathogenesis and efficacy of interventions, the DSS chemical-induced model is the one most extensively used due to its simplicity and how it quickly induces inflammation related to dysbiosis and gut barrier damage [11]. We investigate how dietary kale protects gut barrier integrity and, particularly, how it impacts colon microbiota function and diversity with ultimate protective benefits against the effects of DSS. We systematically analyze changes within each phylum and how that correlates with severity of DSS and HF diet inflammation markers. For our control group, we use a HF diet because this mimics Western society’s diet but does not induce UC. The purpose of this study was not to test effects of a HF diet. DSS-induced colitis does not require a HF diet. In addition, the HF diet was given for only 2 weeks, which is too short a time to induce noticeable phenotype changes. We report that supplementing the HF diet with kale impacts the community structure of the microbiota and its metabolic function, with the most significant changes being attributable to the proportions of proinflammatory LPS- and anti-inflammatory LPS-producing taxa and their downstream metabolic effects.

## 2. Materials and Methods

### 2.1. Animal Study

All animal study procedures were approved by the Institutional Animal Care and Use Committee (IACUC) of the University of Maryland (ref: R-JUL-21-48) approved on July 21st 2021. The study utilized 8-week-old male C57BL/6J mice (*n* = 32) (Jackson Labs, Bar Harbor, ME, USA). The molecular weight of the DSS (MP Biomedicals, Irvine, CA, USA) used was 36–50 kDa. Curly green kale purchased from the local farmers’ market at the University of Maryland was chopped, freeze-dried and powdered for inclusion in the animal food.

Mice were randomized into 4 groups based on body weight to ensure all groups had an equal average starting weight. A sample size of *n* = 8 was used based on a power analysis in our previous animal study on obesity-induced insulin resistance which showed 8 to be a number sufficient to generate statistically significant results. All mice were singly housed in controlled environmental conditions (22 °C) with a 12 h light/dark cycle in shoebox cages containing corncob bedding. After one week of acclimation, mice were switched from the chao diet to a HF diet (45% fat) or a HF diet supplemented with 4.5% kale (*w*/*w* basis) for two weeks. The 4.5% kale was equivalent to 0.11 g per day for a 30 g mouse. Assuming a human consumes 4.1 lbs (1.81 kg) of food per day, that translates into 0.08 kg of kale per day. The kale dose was arbitrary, not based on any prior study, as this was a preliminary study. All diets were formulated to be isocaloric (3961 kcal/kg), as shown in Table 1, and made by the company Research Diets Inc. (New Brunswick, NJ, USA). The amount of insoluble dietary fiber in kale was controlled by adding an equivalent amount of cellulose in the control HF diet. At the end of week 2, exactly 3% (*w*/*v*) DSS was provided via drinking water to 2 groups for 7 days. The control groups were given tap water. The experimental design is illustrated in Table 2. Both food and water were provided *ad libitum*, and consumption was monitored daily. Fresh DSS solution was made every 2 days. Leftover DSS water in the drinking bottles from each experimental group was determined daily over the 7 days.

### 2.2. Disease Activity Index (DAI)

To assess the severity of DSS-induced colitis, the parameters monitored over 7 days were: weight loss, stool consistency, rectal bleeding (presence of blood in stool), dehydration and animal activity. These were scored and used to calculate the DAI over the 7 days that DSS or control tap water was provided using established methods as shown in Wirtz et al. [12].

### 2.3. Euthanasia and Tissue Collection

After 21 days, mice were euthanized by isoflurane inhalation and cervical dislocation. Terminal bleeding was performed by cardiac puncture. All tissues, colon samples and fecal samples were collected and were snap-frozen in liquid nitrogen before being stored at −80 °C. The middle and distal parts of the colon were stored in 10% formalin for histology.

### 2.4. Hematoxylin and Eosin (H & E) Staining

Longitudinal sections of the middle part of the colon were embedded in paraffin and sectioned (4 μm), then stained with hematoxylin and eosin using the standard procedure as shown before in Wirtz et al. [12]. The sections were examined, and histological alterations were scored under a bright-field microscope. The pathological evaluation was performed by a certified pathologist. Histological sections were evaluated based on the extent of immune cell infiltration in the mucosa, submucosa and transmural level and the severity of the epithelial layer damage in the colon.

### 2.5. RNA Extraction and cDNA Preparation

About 50 mg of colon tissue was disrupted in TRIzol with bead beating on the FastPrep^®^-24 (MP Biomedical, Solon, OH, USA) before RNA was extracted and purified using the RNAeasy Mini Kit (Qiagen, German Town, MD, USA). Both RNA quantity and quality were determined using the Qubit 4 Fluorometer (Thermo Fisher Scientific, RockVille, MD, USA). Samples with RNA concentration over 2.3 ng/μL and RNA integrity greater than 7.8 were used for cDNA synthesis using the High-Capacity cDNA Reverse Transcription Kit (Qiagen, German Town, MD, USA) with 1000 ng as starting RNA per sample.

### 2.6. Quantifying the Target Tight Junction Genes and Inflammation Markers by qPCR

The weight of the spleen was determined at euthanasia because an enlarged spleen is a marker of inflammation. Previous studies have shown that the damage to gut barrier integrity is the key pathological characteristic of DSS-induced colitis. Hence, in addition to cytokine markers of inflammation, we evaluated the gene expression of proteins that make up the tight junctions using the validated primer sets (IDT Technologies, Coralville, IA, USA) listed in Table 3. The RT-PCR cycling conditions on the CFX 96 (Bio-Rad, Hercules CA, USA) were 2 min at 50 °C and 2 min at 95 °C, followed by 40 cycles of two-step PCR denaturation at 95 °C for 15 s and annealing extension at 60 °C for 1 min. Triplicate samples contained 10 ng cDNA and 6 μmol/L primers in 2× PowerUp™ SYBR™ Green Master Mix (Thermofisher Scientific, RockVille, MD, USA) in a final volume of 20 μL. A relative amount of target mRNA was normalized to TATA box protein (TPB) levels as an endogenous control gene. Data were analyzed by the 2^−ΔΔCT^ method, and fold difference was calculated between the groups.

### 2.7. Determination of Protein Expression by Western Blot

Protein was extracted by homogenizing 20 mg colon tissue in 1X RIPA buffer supplemented with 1% protease inhibitor and 1 mM PMSF. After determining protein content, samples were denatured for SDS-PAGE gel separation followed by standard immunoblotting. Antibodies used were F4/80 and occludin (both from Cell Signaling Technology, Danvers, MA, USA) and ZO-1 and claudin-1 (Thermo Fisher Scientific, Rockville, MD, USA). Protein expression was normalized by β-actin, and optical densities of protein bands were quantified using ImageJ version 1.53t. Finally, protein expression was calculated as fold difference of the HF-diet control group.

### 2.8. DNA Extraction and Amplification of the 16S Hypervariable Regions

Using the PowerFecal Pro DNA Kit, DNA was extracted from 50 mg colon contents and cleaned up by the DNeasy PowerClean Pro Cleanup Kit (both from Qiagen, Germantown, MD, USA) to remove the PCR inhibitors for downstream sequencing. After determining DNA concentration using the Qubit™ dsDNA BR assay kit, seven hypervariable regions, i.e., V2, V3, V4, V6, V7, V8 and V9, were amplified by PCR using the Ion 16S™ Metagenomics Kit (Thermo Fisher Scientific, Rockville, MD, USA). Amplification products were purified using Agencourt AMPure XP beads (Beckman Coulter, Nyon, Switzerland) on the DynaMag™-2 magnetic rack to remove primer dimers and small mispriming products, washed with fresh 70% ethanol, then eluted into 15 μL of nuclease-free water. The purified PCR product was quantified by the Qubit™ dsDNA HS assay kit before DNA was calculated for library preparation.

### 2.9. Library Preparation, Template Preparation and Sequencing

Libraries were prepared using the Ion Plus Fragment Library Kit (Thermo Fisher Scientific, Carlsbad, CA, USA). After ligating and barcoding each sample and purifying them using AMPure XP beads, library concentration was determined by the Qubit™ dsDNA HS assay kit. After dilution, 10 pM of each sample was used for template preparation, amplified by PCR, purified using fresh 70% ethanol to remove primer dimers and small mispriming products and then eluted into 20 μL of nuclease-free water. After template enrichment on the Ion OneTouch™ 2, template-positive Ion PGM™ Hi-Q™ Ion Sphere™ particles (ISPs) with 400 base pair average insert libraries were used for sequencing on a 530™ Chip using the Ion GeneStudio™ S5 system (Thermo Fisher Scientific, Grand Island, NY, USA).

## 3. 16S rRNA Gene Amplicon Sequencing Analysis

Fastq files from the sequencing run were processed through the QIIME2 pipeline as shown by Bolyen et al. [13] using the DADA2 plugin [14] to determine amplicon sequence variants (ASVs). Data were rarefied to a sampling depth of 10,000 reads per sample, and a phylogenetic tree was built with FastTree. Taxonomic classification was performed using the SILVA v138 database [15]. We performed multivariate testing between treatment groups and further performed post hoc comparisons when significant differences were observed. We determined alpha diversity indices: observed ASVs (a count of unique ASVs in each sample), Shannon diversity (to account for both abundance and evenness of the sequence variants present) and Faith PD, which uses phylogenetic distance (branch length of tree) to calculate the diversity. Beta diversity was calculated based on the Bray–Curtis distance matrix and visualized using principal component analysis (PCA) as implemented in QIIME2.

### 3.1. Linear Discriminant Analysis

Using the Galaxy workflow (https://huttenhower.sph.harvard.edu/galaxy accessed on 20 November 2022) we determined the taxa most likely to explain the differences between the four groups using linear discriminant analysis effect size (LEfSe), which utilizes a non-parametric Kruskal–Wallis rank-sum test to assess differential features with significantly different abundances between assigned taxa and performs LDA to estimate the effect size of each sequence variant as reported by Segata et al. [16]. LDA scores ranking differential taxa are displayed on a bar chart according to their effect size. A significant alpha level of 0.05 and an effect size threshold of a three times greater difference were used for displaying results.

### 3.2. PICRUSt2

To predict the key bacterial metabolic pathways that account for phenotype differences between the groups, phylogenetic investigation of communities by reconstruction of unobserved states (PICRUSt2) was performed from sequencing data, as shown before by Douglas et al. [17]. Fastq files were used as input files for the PICRUSt2 pipeline. Metabolic pathways were assigned based on the Kyoto Encyclopedia of Genes and Genomes (KEGG) Ortholog (KO) database. Read abundance data for all predicted pathways were converted to relative abundance and analyzed on the Galaxy server (https://huttenhower.sph.harvard.edu/galaxy/, accessed on 20 November 2022) for LEfSe using an LDA score 3.0 as a threshold level to determine pathways taxa most likely to explain differences between the four groups. The heatmap for the PICRUSt2 data was prepared using R software (version 4.0.2).

### 3.3. Determination of LPS and LPB in Serum

Endotoxin levels were determined by quantifying serum LPS levels using a mouse sandwich ELISA kit (My Biosource, San Diego, CA, USA) and lipopolysaccharide binding protein (LBP) ELISA kit (Abcam, Cambridge, UK) following the manufacturers’ provided protocols.

### 3.4. Quantification of genus Turicibacter by qPCR

To validate the findings by NGS that the kale vegetable elicited increases in the abundance of genus *Turicibacter*, and specifically the species *Turicibacter sanguinis*, we quantified this genus and species by qPCR. The primers used for *Turicibacter* were F: 5′ CAG ACG GGG ACA ACG ATT GGA 3′ and R: 5′ TAC GCA TCG TCG CCT TGG TA 3′, as reported by Kable et al. [18], and the primers for *Turicibacter sanguinis* were F: 5′ GGGTTTGCTGATGCCGC 3′ and R: 5′ CCCAAATTTATCAACTACTGCTGTAATAAT 3′, as reported by Lynch et al. [19]. Both primers were produced by Integrated DNA Technologies (Coralville, IA, USA). Genomic DNA from pooled samples in each treatment group was used as a template to prepare amplicon DNA by PCR on the CFX 96 Touch^TM^ System (Bio-Rad, Hercules, CA, USA). Standard curves were generated by serial 10-fold dilutions of amplicon DNA with nuclease-free water. Standards and samples (diluted 20-fold) were analyzed by SYBR Green qPCR Assay. Cycling conditions were 50 °C for 2 min, 95 °C for 2 min, 40 cycles of 95 °C for 15 s, followed by primer annealing at 60 °C for 1 min. The Cqs for non-template controls were used to adjust all values. Amplicon quantities (gene copies per microliter) versus cycles-to-threshold standard curves were used to determine quantities of target species. The molar concentrations were converted into gene copies per microliter using the formula shown by Oldham and Duncan [20]. This assumes the average molecular mass of the double-stranded DNA base pairs is 6.6 × 10^11^ ng/mol, and the Avogadro’s number of copies per mole is 6.022 × 10^23^. Amplicon length of *Turicibacter* was 254 bp.

Copies per microliter were thus calculated as:

$$\begin{array}{l} \left[\mathrm{DNA}\;\mathrm{concentration}\;(\mathrm{nanograms}\;\mathrm{per}\;\mathrm{microliter})]\times [6.02\times 10^{23}\;(\mathrm{copies}\;\mathrm{per}\;\mathrm{mole})\right]\\ {}[\mathrm{amplicon}\;\mathrm{length}\;(\mathrm{bp})\times 6.6\times 10^{11}\mathrm{ng}/\mathrm{mol}]. \end{array}$$

### 3.5. In Vitro Tests Using Kale Extract

The effect of the ethanolic extract of kale (70% ethanol) on P-LPS-induced inflammation was determined in RAW 264.7 murine macrophages (ATCC, Manassas, VA, USA). Cells were grown and maintained in DMEM supplemented with 10% FBS at 37 °C and 5% CO_2_. Two major components of kale, i.e., sulforaphane and beta-carotene, were also tested to compare performance with that of the whole extract. Cells were pretreated with 100 μg/mL ethanolic extract, 100 ng/mL sulforaphane or 20 ng/mL beta-carotene (both from Sigma Aldrich, St. Louis, MO, USA). After 4 h, they were stimulated with 100 ng/mL P-LPS from *E. coli* O111:B4 (Sigma-Aldrich, St. Louis, MO, USA) for 24 h. After collection of cells, RNA was extracted, quantified and reverse transcribed and the gene expression of inflammatory markers determined by qPCR as shown for the colon tissue.

## 4. Statistical Analysis

Effects of the treatments (HF, HFKV, HF-DSS, HFKV-DSS) were compared by one-way ANOVA in GraphPad 9.0. Effects were considered significant at *p* < 0.05 and expressed as means ± SE. When significant differences were observed, the Tukey–Kramer test of multiple comparisons was performed. In cell culture, effects of treatments were similarly compared between the control, LPS only, LPS + ethanolic extract, sulforaphane or beta-carotene. For gut microbiota data, statistical significance for analysis of alpha and beta diversity was determined with a variety of tests in QIIME2. The relative abundance values of different taxa were converted to percentages.

## 5. Results

### 5.1. Determination of DAI Score Parameters

**Figure 1:**
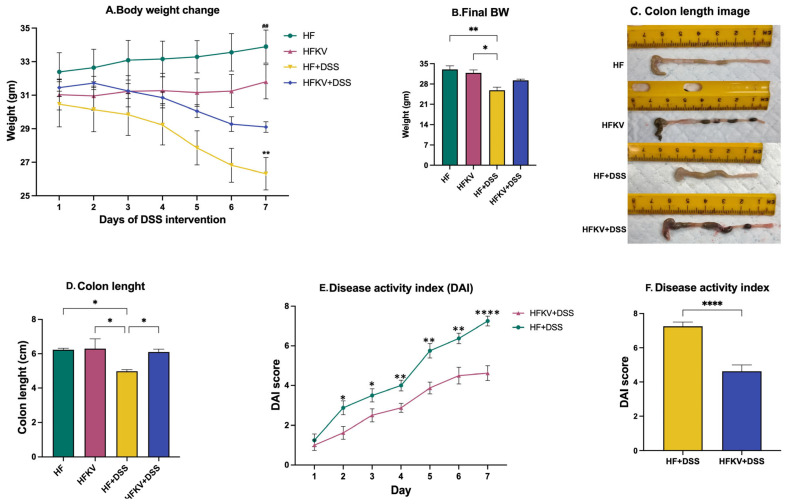
Kale supplementation attenuates markers of the DAI score and prevents colon shortening. Body weight was determined daily during DSS administration. The colon length was determined at euthanasia. The DAI score was calculated based on weight loss, stool consistency, bleeding level, activity of the animal and water consumption. (**A**,**B**) Body weight trends over 7 days and at the end of 7 days of DSS administration. (**C**) Representative pictures of colons displaying the length at euthanasia. (**D**) The calculated average colon length. (**E**) DAI score over 7 days of DSS administration. (**F**) Mean DAI score after 7 days. Results are shown as mean ± SEM. *p*-values * = *p* < 0.05, *n* = 8; ** = *p* < 0.005, *n* = 8; **** = *p* < 0.0001, *n* = 8. ^##^ = *p* < 0.005, *n* = 8.

Despite food intake and water intake not being different during the first two weeks or during the 7-day period of DSS administration (Supplementary Figure S1), the body weight of mice in the HF-DSS treatment group notably declined compared with that of the group supplemented with kale, HFKV-DSS (*p* < 0.001; Figure 1A,B). No weight loss was observed in the HF and KFKV groups. The normal length of the colon was decreased in the HF-DSS group. Kale supplementation before DSS prevented this; no reduction in colon length was observed in the HFKV-DSS group (Figure 1C,D). Overall, the calculated DAI score was significantly lower in the HFKV-DSS group compared to in the HF-DSS group. Kale supplementation thus attenuated the severity of the DAI scores (Figure 1D,E).

### 5.2. Effects of Kale Supplementation on Histopathological Changes and Splenomegaly

**Figure 2:**
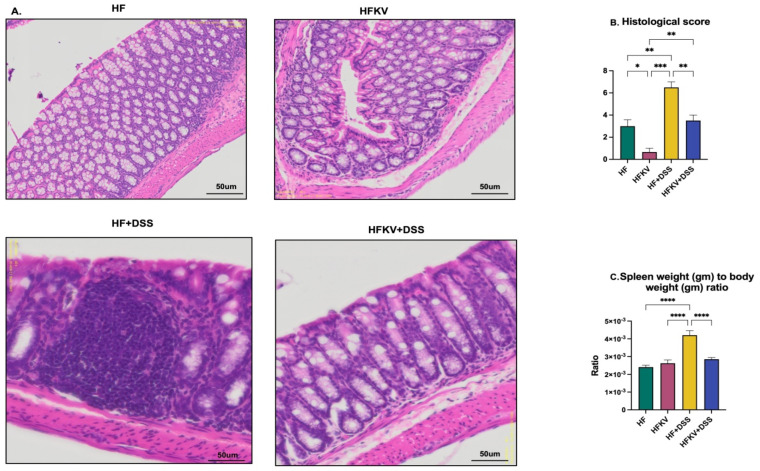
Kale supplementation protects against histopathological damage and enhances gut integrity. Longitudinal sections of the colon embedded in paraffin were sectioned and then stained with H & E. The histological score was calculated based on extent of inflammatory cell infiltration in the mucosa, submucosa and transmural level and the damage in epithelial layers. The weight of the spleen was determined at euthanasia. (**A**) Representative H & E staining images of each group. (**B**) Mean histological score of each group. (**C**) Ratio of weights of the spleen and animal weight. Results are shown as mean ± SEM. *p*-values * = *p* < 0.05, *n* = 8; ** = *p* < 0.005, *n* = 8; *** = *p* < 0.001, *n* = 8. **** = *p* < 0.0001, *n* = 8.

Mice showed notable distortion of crypts and histological damage in the mucosa and submucosa after the DSS administration. Kale supplementation protected against the severe histological damage of the colon (Figure 2A), and structural organization was at a normal level. In fact, the histological score in the KFKV-DSS mice was not different from that of mice fed only the control diet as the ultrastructure was intact. Furthermore, the histological score in control mice fed kale (HFKV group) was better than that of the control mice fed the HF diet only (Figure 2B). This implies that even in mice not given DSS, kale supplementation improved the condition of the colon. The weight of the spleen significantly increased in the HF-DSS group, and kale supplementation prevented this (Figure 2C).

### 5.3. Effects of Kale Supplementation on Gut Barrier Integrity

**Figure 3:**
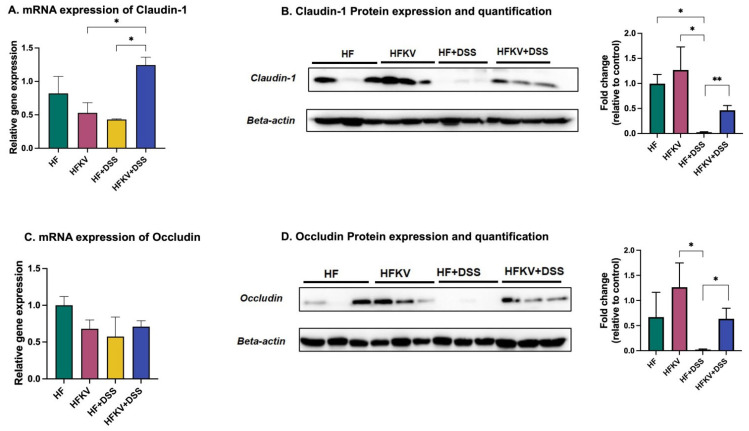
Kale supplementation promotes gut barrier integrity. Gene and protein expression of tight junction proteins was determined by RT-qPCR and Western blotting of colon tissue. (**A**) m-RNA expression of claudin-1. (**B**) Protein expression and quantification of claudin. (**C**) mRNA expression of occluding. (**D**) Protein expression and quantification of occluding. Results are shown as mean ± SEM. *p*-values * = *p* < 0.05, *n* = 3; ** = *p* < 0.005, *n* = 3.

Compared with the control HF group, supplementation with kale significantly enhanced the mRNA and protein expression of claudin-1 and protein expression of occludin even in mice not given DSS, i.e., the HFKV group. The DSS regime reduced mRNA and protein expression of claudin-1 and occludin (almost undetectable by Western blotting). Supplementation with kale prevented DSS from obliterating the gene and protein expression of claudin and occludin (*p* < 0.001; Figure 3). The full, uncropped Western bolts are shown in Supplementary Figure S2A (panels A–D). The mRNA expression of MUC-2 and ZO-1 was not significantly changed by kale supplementation. Overall, this finding supports the fact that kale prevents DSS-induced gut barrier damage. Furthermore, kale enhanced expression of the tight junction proteins even in mice not given DSS.

### 5.4. Effects of Kale Supplementation on Inflammatory Responses

**Figure 4:**
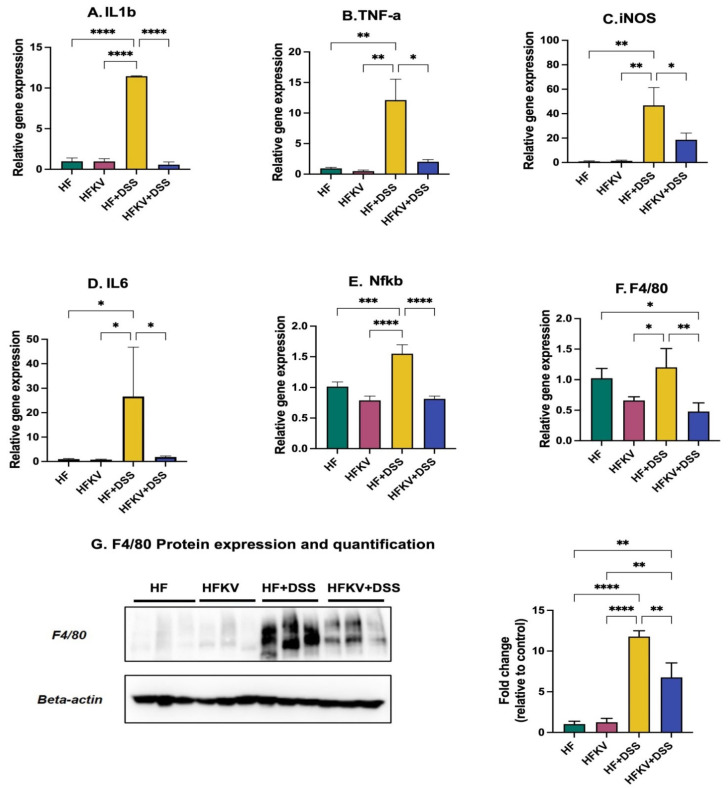
Effects of kale supplementation on inflammatory responses. RNA and protein were extracted from colon tissue, and gene expression of inflammatory markers was determined by qPCR and Western blotting. (**A**–**F**) Gene expression of inflammatory markers. (**G**) Protein expression of F4/80 marker of macrophages. Results are shown as mean ± SEM. *p*-values * = *p* < 0.05, *n* = 3; ** = *p* < 0.005, *n* = 3; *** = *p* < 0.001, *n* = 8; **** = *p* < 0.0001, *n* = 8.

The increase in weight of the spleen caused by DSS is a marker of inflammation (Figure 2C), and including kale in the diet prevented this (*p* < 0.001; Figure 2C). Administration of DSS induced very significant increases in expression of TNFa, iNOS, IL-6, iL-1b and NfKB compared to the control HF diet (Figure 4). Supplementation with kale lowered the expression of these genes to levels not different from those in the control HF and HFKV groups (*p* < 0.01; Figure 4). The gene and protein expression of F4/80, the marker for macrophage infiltration, was upregulated by DSS, and supplementation with kale attenuated this expression (Figure 4G). The full, uncropped Western blot image is shown in Supplementary Figure S2B.

### 5.5. Effects of Kale Supplementation on Gut Microbiota Composition and Diversity

#### 5.5.1. Rarefaction

The rarefaction curves for all samples reached a plateau, indicating that the sequencing depth was sufficient to detect the majority of ASVs in each sample and capture the microbial diversity (Supplementary Figure S3). The rarefaction curves indicated that DSS reduced richness (fewer ASVs), and supplementing the diet with kale attenuated this regardless of sequencing depth. Even in mice not given DSS, kale supplementation enhanced richness (Supplementary Figure S3A).

#### 5.5.2. Alpha Diversity Measures

**Figure 5:**
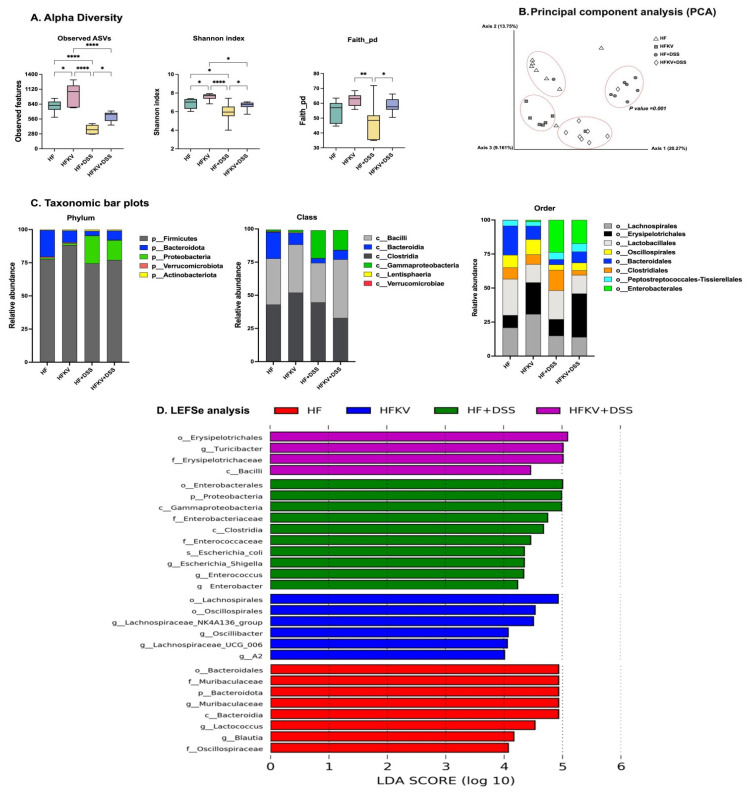
Effects of kale supplementation on the gut microbiome structure and diversity. Bacterial DNA was extracted from colon contents and used to sequence the 16S gene. Fastq files were used for bioinformatics analysis in QIIME2 to determine diversity and taxa abundance. Analysis by LeFSe was used to determine taxa that contributed most to the differences between groups. (**A**) alpha diversity matrices. (**B**) Beta diversity based on the Bray–Curtis distances between samples. (**C**) Abundance of bacterial taxa at phylum, class and order levels. (**D**) Linear discriminant analysis (LDA) scores. Results are shown as mean ± SEM. *p*-values * = *p* < 0.05, *n* = 8; ** = *p* < 0.005, *n* = 8; **** = *p* < 0.0001, *n* = 8.

*All alpha diversity measures that we determined showed that DSS lowered the diversity of the microbiota.* The microbiota of HF-DSS mice had significantly lower alpha diversity with the number of ASVs, Faith PD and Shannon indices showing significantly lower values than those of mice fed a HF diet. Supplementation with kale attenuated this DSS-induced decrease (*p* < 0.001; Figure 5A). Further analysis showed that all alpha diversity measures were significantly higher in HFKV mice compared to in mice fed a HF diet only (*p* < 0.001; Figure 5A).

#### 5.5.3. Beta Diversity

We performed pairwise tests for differentiation in a centroid location, i.e., the mean position of all the samples within each group (PERMANOVA). The one-way PERMANOVA non-parametric test of significant difference between groups, based on the Bray–Curtis distance, showed significant differences between all four groups but not within groups (Supplementary Figure S3B,C). Figure 5B visualizes them in a PCA plot. The clustering was mainly based on treatment group, indicating a clear, distinct impact of each of the four treatments based on diet and DSS.

#### 5.5.4. Comparative Analysis of the Gut Microbiota Taxa Composition

**Figure 6:**
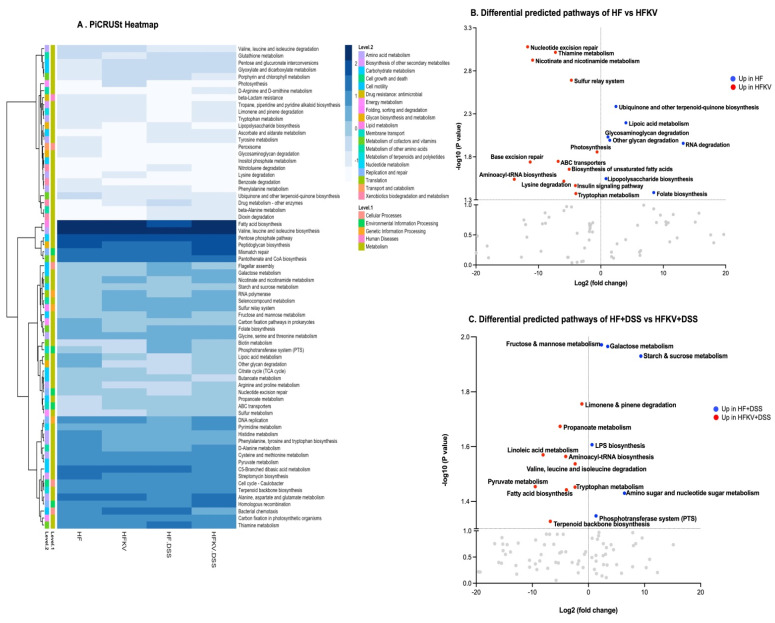
Effects of kale supplementation on predicted bacterial gene pathways. Fastq files were input for analysis in PICRUSt2 to predict relative intensities of various bacterial pathways. The data on pathways identified were analyzed by LEfSe to identify pathways that most likely accounted for differences among the groups. (**A**) A heatmap comparing bacterial pathways and their intensity. (**B**) Volcano plot showing key predicted bacterial pathways that were different between HF and HFKV groups. (**C**) Volcano plot showing key predicted bacterial pathways that were different between HF-DSS and HFKV-DSS groups.

**Figure 7:**
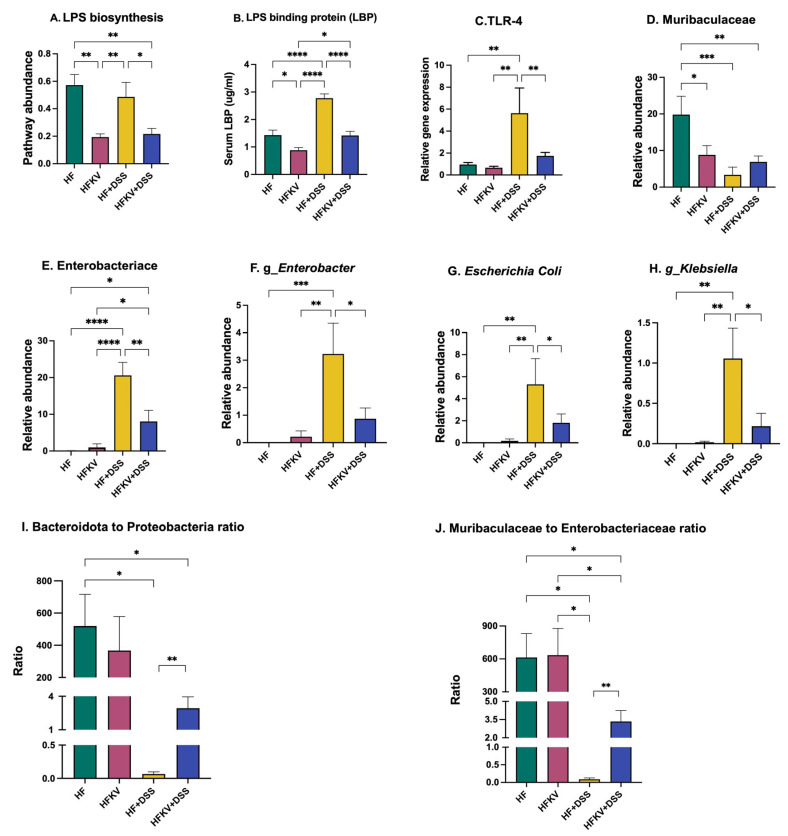
Effects of kale supplementation on LPS responses and abundance of LPS-producing taxa. Relative abundance of LPS-producing bacteria and pathway abundance of LPS-producing genes were calculated from 16S sequencing data and PICRUSt2 output. Serum LPB expression was determined by ELISA kit while TLR4 expression in colon tissue was determined by qPCR. (**A**) LPS biosynthesis pathway abundance. (**B**) LPB protein expression in serum. (**C**) TLR4 gene expression in colon tissue. (**D**) Relative abundance of A-LPS-producing taxa. (**E**–**H**) Relative abundance of P-LPS-producing taxa. (**I**,**J**) Ratio of A-LPS and P-LPS-producing bacterial taxa; *Bacteroidota*:*Proteobacteria* and *Muribaculaceae*:*Enterobacteraceae*. Results are shown as mean ± SEM. *p*-values * = *p* < 0.05, *n* = 8; ** = *p* < 0.005, *n* = 8; *** = *p* < 0.001; *n* = 8. **** = *p* < 0.0001, *n* = 8.

The patterns observed in the alpha and beta diversity analysis were confirmed after taxonomic assignments as more taxonomic groups were observed in the mice fed kale. Fewer taxa were observed in mice given DSS, and kale supplementation attenuated this (Figure 5). Predicted bacterial pathways based on the taxa identified in each group are shown in Figure 6. Qualitative analysis showed that the most significant change induced by DSS was an increase in phylum Proteobacteria. Proteobacteria were present at very low proportions in the HF and HFKV groups at 1% and 1.4%, respectively. Administration of DSS increased proportions of these taxa to 20%. Kale supplementation attenuated this increase to 13%. Most of the bacteria in this phylum were from class Gammaproteobacteria, order Enterobacteriales, with only family Enterobacteriaceae detected (Figure 5C and Figure 7E–H).

*Bacteroidota* consisted solely of the class Bacteroidia, order Bacteroidales and specifically family Muribaculaceae. The control HF-fed mice had the highest number of these taxa. Supplementation with kale reduced numbers by one-fold. Mice given DSS had much lower numbers of these taxa, and kale did not significantly change the proportions (Figure 5C and Figure 7D).

**Figure 8:**
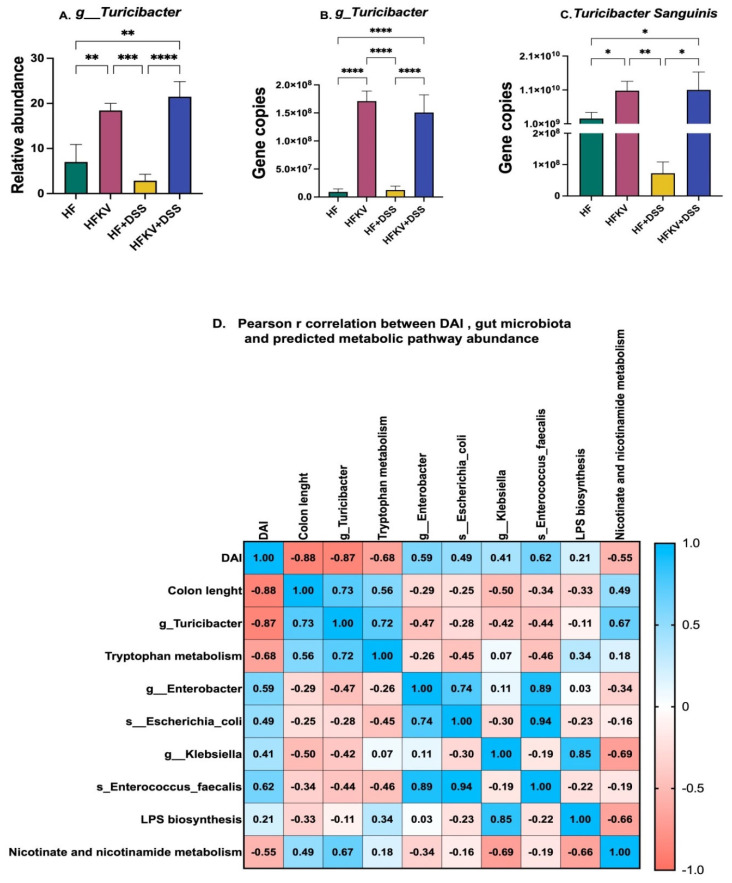
Spearman correlation between key bacterial taxa or pathways impacted by kale and the DAI score and colon length. Relative abundance of genus *Turicibacter*, *Enterobacter* and *Klebsiella* was calculated from 16S sequencing data and validated by qPCR. A correlation of bacterial pathways, taxa and the DAI score was performed in GraphPad. (**A**) Abundance of genus *Turicibacter* after NGS. (**B**) Gene copies of *Turicibacter* after Qpcr. (**C**) Gene copies of *Turicibacter* sanguinis after Qpcr. (**D**) A correlation of bacterial gene pathways, key taxa and the DAI score. Results are shown as mean ± SEM. *p*-values * = *p* < 0.05, *n* = 8; ** = *p* < 0.005, *n* = 8; *** = *p* < 0.001, *n* = 8; **** = *p* < 0.0001, *n* = 8.

Firmicutes constituted the largest taxa with over 74–88% of the representation in all mice, with class Clostridia and Bacilli forming the core. This phylum was the most diverse in terms of lower-level taxa. Supplementation with kale increased the proportions of Firmicutes. Breaking down the data at order levels revealed that it was the order Erysipelotrichia that significantly increased. Among this order, the family/genus whose representation was increased by kale supplementation was Turicibacteraceae/*Turicibacter*. It increased by two-fold in HFKV-fed compared to HF-fed mice and increased by more than six-fold in HFKV-DSS mice compared to in HF-DSS mice (Figure 5C and Figure 8A–C). Thus, kale increased Turicibacter both in the control group and the DSS treatment group. Follow up by qPCR showed that most of the order Turicibacter was composed of the species *Turicibacter sanguinis* (Figure 8A–C).

#### 5.5.5. Linear Discriminant Analysis Effect Size (LEfSe)

The LEfSe algorithm was used to identify the key bacterial taxa that accounted for the differences between the groups, particularly to estimate the effect size of taxa that were significantly different in representation and to rank them. At a threshold of 4.0 for the logarithmic LDA score, the abundance of 28 taxa was different between the four groups and accounted for discriminative features between the mice (Figure 5D). A *p*-value of less than 0.05 was considered significant in a non-parametric Kruskal–Wallis rank test. The LEfSe output was in agreement with the abundances shown in the qualitative plots of taxa abundances (Figure 5C), showing that the abundance of bacteria from order Enterobacteriales and order Erysipelotrichia accounted most for the discriminative features between the treatments.

### 5.6. Predicted Metabolic Functions

PICRUSt2 predicted a total of 150 functional pathways by comparison against KEGG orthologs. Data were transformed to relative abundance, and differences between diets are presented in Figure 6. The LEfSe analysis at a 2.5 threshold level and at a *p*-value of 0.05 in a non-parametric Kruskal–Wallis rank-sum test showed that 38 pathways accounted for the discriminative features between the four groups (Supplementary Figure S4).

The top 10 pathways that were significantly higher in the microbiota of HFKV mice compared to in that of HF-fed mice were aminoacyl-tRNA biosynthesis, biosynthesis of unsaturated fatty acids, insulin signaling pathway, nicotinate and nicotinamide metabolism, nucleotide excision repair, photosynthesis, sulfur relay system, thiamine metabolism, tryptophan metabolism and lysine degradation, as illustrated in the volcano plot in Figure 6B. The top 10 pathways that were significantly higher in the microbiota of HFKV-DSS mice compared to in that of HF-DSS mice were limonene and pinene degradation, propanoate metabolism, linoleic acid metabolism, valine leucine and isoleucine degradation, aminoacyl t-RNA biosynthesis, D-arginine and D-ornithine metabolism, tryptophan metabolism, pyruvate metabolism, fatty acid biosynthesis and terpenoid backbone biosynthesis, as shown in the volcano plot in Figure 6C. The LPS synthesis pathway was higher in HF-DSS mice compared to in HFKV-DSS mice.

### 5.7. Effects of Kale Supplementation on LPS-Producing Bacteria and the LPS-Producing Pathway

An analysis of key bacterial genes by PICRUSt2 revealed that DSS induced an increase in the gene expression of bacterial genes that produce LPS, but this increase was attenuated in the microbiota of mice fed kale (Figure 7A). Kale lowered expression of bacterial genes that produce LPS in both HF-fed mice and those fed HF and then given DSS. The expression of TLR4 in colon tissue was increased by DSS, and kale supplementation prevented this increase (Figure 7C). The protein TLR4 is the sensing receptor for LPS and binds other endogenous molecules produced because of tissue injury to induce a proinflammatory response. Results from the ELISA test for LPS in serum were inconclusive because our samples were degraded. However, the amount of LPS binding protein (LBP) significantly increased due to the DSS regime. Kale attenuated the expression of LBP in mice given DSS to levels not different from those with the HF control diet (*p* < 0.001, Figure 7B). A further analysis of sequencing data showed that among Gram-negative LPS-producing taxa, at family level, DSS reduced *Muribaculaceae* while increasing the representation of Enterobacteriaceae. Thus, DSS induced a shift in the intestinal microflora towards proinflammatory Gram-negative bacteria. Specific genera increased were *Enterobacter* and *Klebsiella* (Figure 7D–H). Supplementation with kale before the DSS regime attenuated the increase in representation of Enterobacteriaceae, *Enterobacter* and *Klebsiella* (*p* < 0.05; Figure 7D–H). Because the gut microbiota is a reservoir for LPS, and specifically type PF LPS based on its toxicity, we determined the ratio of *Bacteroidota*:*Proteobacteria*, and, further, at family level, *Muribaculaceae*:Enterobacteriaceae was determined as a measure of LPS type and its potential effects.

### 5.8. Effects of Kale Supplementation on Genus Turicibacter and Tryptophan Metabolism Pathway

16S sequencing data analysis revealed a major effect of kale supplementation on the abundance of genus *Turicibacter* (Figure 8A). Specifically, kale induced an increase in *Turicibacter* from less than 7% to over 18% of total bacteria in control HF-fed mice and from 3% to 22% of total bacteria in mice given DSS. We therefore performed qPCR using primers specific to this genus to validate this finding. Data from qPCR data confirmed the findings from the 16S sequencing. Specifically, the gene copies of genus *Turicibacter* and the species *Turicibacter sanguinis* significantly increased in both groups fed kale (Figure 8B,C). Bacteria from *Turicibacter* have been linked to tryptophan metabolism. PICRUSt2 analysis revealed that the tryptophan metabolism pathway was significantly increased by kale supplementation. The Spearman correlation revealed a strong positive correlation between the tryptophan metabolism pathway and *Turicibacter* abundance (R^2^ = 0.72) (Figure 8). The correlation further revealed a strong negative correlation between the DAI core and *Turicibacter* abundance (R^2^ = −0.87) and similarly between the DAI core and the tryptophan metabolism pathway (R^2^ = −0.68) (Figure 8D).

### 5.9. Effect of Kale Extract on LPS-induced Inflammation in RAW 264.7 Macrophages

**Figure 9:**
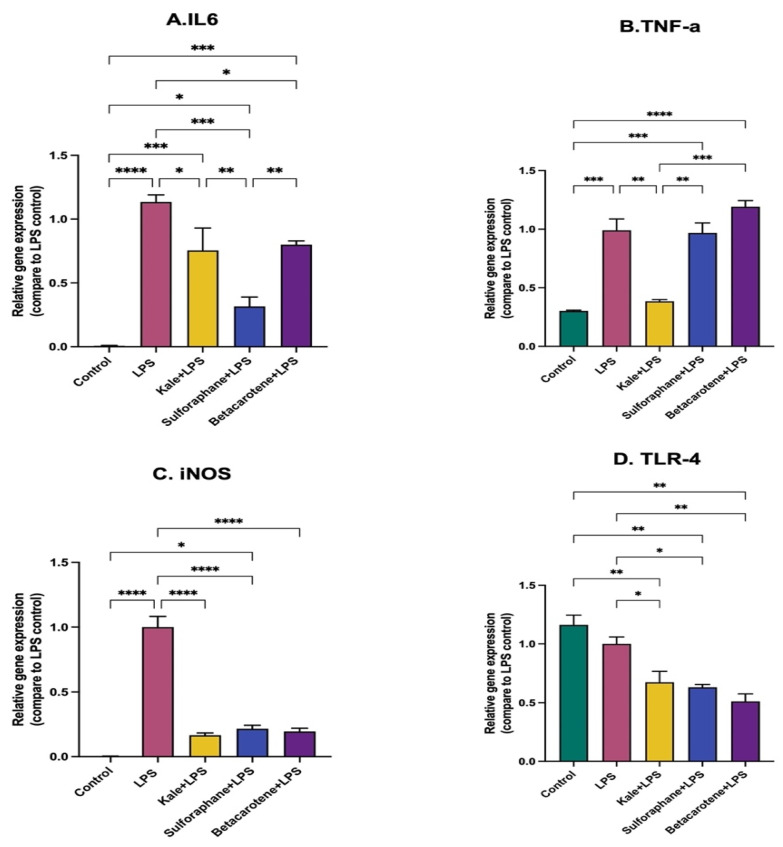
Effects of kale extract and bioactive compounds of kale on LPS-induced inflammation in RAW 264.7 macrophages. RAW 264.7 macrophages were treated with an extract of kale, sulforaphane or beta-carotene before stimulation by P-LPS. Gene expression of inflammatory cytokines was determined by qPCR. (**A**–**D**) The relative gene expression of IL6, TNF-a, iNOS and TLR4 among different treatment groups. Results are shown as mean ± SEM. *p*-values * = *p* < 0.05, *n* = 3; ** = *p* < 0.005, *n* = 3; *** = *p* < 0.001, *n* = 3, **** = *p* < 0.0001, *n* = 3.

The role of kale in attenuating the inflammation was further evaluated by treating RAW 264.7 macrophages with an ethanolic extract of kale or two major bioactive compounds of kale, i.e., sulforaphane and beta-carotene, before stimulation with P-LPS. The P-LPS induced significant increases in IL-6, iNOS and TNFa. Kale extract, sulforaphane and beta-carotene all significantly prevented or attenuated increases in expression of IL6 and iNOS. However sulforaphane performed better than the extract in attenuating IL-6, while the extract performed better at attenuating TNFa (Figure 9A,B). Beta-carotene had no effect on TNFa expression. All three downregulated the expression of TLR4 compared to the LPS-stimulated and control cells (Figure 9D).

## 6. Discussion

Because it is established that dietary interventions play a key role in protecting against severe or chronic inflammation, we investigated the protective effects of kale, a ‘functional food’ that is often on the list of ‘the most healthy foods’. Kale is widely cultivated and incorporated in traditional meals, especially in the Mediterranean area, and is now becoming a popular vegetable in the United States. Given that inflammation arising from LPS effects is associated with many different diseases, research into how this ‘super food’ may be protective is justified. In this study, we successfully confirmed the protective effects of kale supplementation in a model of severe lower gut inflammation and gut barrier damage. Specifically, our results showed that kale attenuates DSS-induced gut microbiota dysbiosis to an extent sufficient to prevent the manifestation of typical DSS-induced molecular, histological and physical markers of acute inflammation associated with IBD.

The phytochemistry of cruciferous vegetables including kale has been widely studied. The benefits of kale are attributed to its abundant glucosinolates, polyphenols, carotenoids, terpenoids and various indole derivatives [21,22]. Kale is also rich in carotenoids and potassium [23]. In the current study, we focused on assessing the impact of the whole vegetable as a protective functional food against the inflammation associated with P-LPS production and translocation but did not analyze the phytochemical composition. One key initial finding was the ability of kale to protect against the typical histological markers of inflammation in the mucosa and submucosa during and after DSS administration. Granuloma was markedly absent in the histology samples from mice supplemented with kale before DSS was administered (Figure 2A). Formation of granulomas is a distinctive sign of chronic inflammation produced in response to a persistent inflammatory stimulus from infectious, autoimmune, toxic chemical injury, allergic or neoplastic conditions. Granulomas result from infiltration of immune cells such as mononuclear leukocytes, specifically macrophages at the site of the infection or inflammation, to eliminate the pathogen [24]. These immune cells clump together into tiny nodule structures, as seen in the histology samples from the HF-DSS group (Figure 2A). This result was further confirmed by the gene expression and protein expression of F4/80. Giving DSS significantly increased this protein in colon tissue, but samples from mice supplemented with kale before DDS had significantly lower expression (Figure 4F,G). F4/80 is a marker for populations of mouse tissue macrophages that originate from embryonic progenitors [25]. The overall histological score was attenuated by kale supplementation, and it was particularly striking that even in control mice not given DSS, kale improved the histology score (Figure 2B), confirming its benefits against both the acute inflammation and low-grade inflammation that may be induced by a HF diet. However, in this study, because the HF diet was the control diet, the extent of inflammation was not measured.

Although not a direct measure of inflammation, spleen weight is a correlation measure of inflammation [11]. As a disease progresses, the resulting immune response leads to an increase in immune cells, and thus an increase in spleen weight is indicative of the robust immune response present. While DSS increased spleen weight by about 30%, no weight gain was observed in the spleens from mice supplemented with kale before DSS administration (Figure 2C), indicating a much-subdued immune response. A shortened colon length and increased DAI score are important inflammation-associated parameters in DSS-induced colitis mouse models. No reduction in colon length was observed in mice fed with kale before the DSS regime (Figure 1). This was further demonstrated by qPCR analysis of inflammatory markers IL-1B, TNFa, iNOS, IL-6 and NfkB, which were drastically increased by DSS administration, but no increase in any of them occurred in samples from mice fed kale before DSS administration (Figure 4). All these results are evidence of the protective effect of kale against inflammation.

Because DSS destroys intestinal barrier function and results in intestinal permeability that substantially exceeds that typically observed in humans, it is impossible to evaluate therapies that restore barrier integrity in this model [26]. However, our study was not about finding a therapy for UC but rather focused on prevention, studying kale as a protective food against destruction of intestinal barrier function. In Figure 3, we show that kale supplementation before the DSS regime significantly prevented the downregulation of the gene and protein expression of tight junction proteins claudin and occludin. Mice that were not fed with kale experienced severe destruction of the gut barrier, evidenced by downregulation in the expression of these proteins. Furthermore, kale enhanced expression of claudin and occludin and thus enhanced intestinal barrier function even in mice not given DSS, further reinforcing its properties as a health promoting food. It is unclear why expression of ZO-1, another tight junction protein, was not changed by kale.

Alterations in the composition and function of the gut microbiota are linked to the pathogenesis of inflammation. A loss or gain of certain bacterial taxa may trigger an immune response in the colon. In germ-free mice, immune-mediated colitis is largely absent, although reductions in barrier function occur at higher DSS concentrations [27,28]. One important finding from this study was that the reduction in microbial diversity by DSS is accompanied by lower proportions of Gram-positive and higher proportions of Gram-negative bacteria (Figure 5). This is an observation commonly reported in IBD patients [29]. Based on the Bray–Curtis distance and cluster analyses, the microbiome from mice given DSS grouped separately from that of mice supplemented with kale before the DSS regime, indicating that the dominant species were different (Figure 5B). The gut microbiota of mice fed the HFKV and HFKV-DSS diets grouped closer together, indicating that the dominant species were similar. Thus, mice fed kale had similarities in microbial composition irrespective of DSS (Figure 5B). The strong positive correlation between the structure of the bacterial community with colon length and DAI score is evidence of the close links between bacterial community structure and the severity of colitis (Figure 8C). A shift towards a proinflammatory intestinal microbiota occurred with DSS. This shift was characterized by an over 20-fold increase in the representation of Enterobacteriaceae (Figure 5 and Figure 7), a finding that is in agreement with previous studies in mice [30,31,32] and also in humans who have IBD [33]. Supplementation with kale before the DSS regime prevented this proinflammatory shift of the microbiota but instead increased the overall diversity, and specifically representation of *Muribaculaceae*, which is home of the taxa known to produce anti-inflammatory LPS [8,9,10] and Gram-positive taxa that are not LPS producers but whose role in LPS signaling is unknown.

The increase in Enterobacteriaceae in the HF-DSS group was accompanied by an increase in circulating LBP protein, the carrier of P-LPS. LBP is a biomarker of both the amounts of LPS entering circulation and the resultant innate immune response. Similarly, the increase in Enterobacteriaceae in the HF-DSS group was accompanied by increases in colon expression of TLR4, the sensing receptor for P-LPS (Figure 7), indicating activation of inflammatory signaling. When P-LPS is recognized by TLR4, a signaling cascade leading to the transcription of inflammatory cytokines and chemokines is triggered to initiate an innate response to eliminate pathogens and attract immune cells [29,30]. While P-LPS does not damage the gut epithelium on the luminal side, it induces toxicity once it reaches the basal side, which has exposure to the deeper tissues [33]. It is notable that although the prominence of the LPS synthesis pathway in the gut microbes (as determined by PICRUSt2) was not different between the control HF group and the HF-DSS group (Figure 7A), LBP and TLR4 were enhanced only in the HF-DSS group (Figure 7B,C). This indicates that the LPS produced in the control HF group and the LPS produced in the HF-DSS group resulted in different signaling effects. In the control HF group, order *Bacteroidales*, particularly *Muribaculaceae*, was the dominant Gram-negative taxa. Bacteria in this taxon have been shown to produce A-LPS whose lipid A moiety is dephosphorylated and deacylated. On the other hand, the HF-DSS group had phylum *Proteobacteria*, particularly order Enterobacterales, as the dominant Gram-negative bacteria, and this produces P-LPS whose lipid A moiety is fully phosphorylated and fully acylated and will bind to TLR4 and hence trigger the inflammatory signaling pathway [8,9,10]. P-LPS, after being recognized and bound to TLR4, will enter circulation and bind to and trigger more expression of LBP, as shown in Figure 7B.

There was no difference in circulating LBP levels and colon TLR4 expression in mice fed the control HF diet and those fed kale before DSS administration (HFKV-DSS), indicating that the LPS produced was overall anti-inflammatory or immunosuppressive since it did not trigger increased TLR4 signaling. Kale supplementation attenuated the DSS-induced shift in the intestinal microflora towards proinflammatory, Gram-negative Enterobacteriaceae (Figure 7). The ratio of Gram-negative A-LPS- and P-LPS-producing taxa (*Bacteroidota*:*Proteobacteria*) was reduced drastically by DSS (Figure 7I,J), and supplementation with kale attenuated it. While the impact of kale on this ratio seemed modest, it was sufficient to retain a balanced immune function, as evidenced by the inflammation marker readouts in Figure 4. Alternatively, the phytochemical compounds of kale may promote secretion of enzymes that dephosphorylate or deacylate P-LPS into A-LPS that is unable to trigger TLR4 signaling. However, this remains to be tested and will be our next step in testing the impacts of kale as a functional food. In vitro studies in macrophages revealed that the whole kale extract performs as well as purified sulforaphane in preventing cytokine secretion in response to LPS stimulation. However beta-carotene did not perform as well as the extract. However, it is unclear whether the extract or sulforaphane does so by changing the LPS structure or by impacting the cells. However, the extract, sulforaphane and beta-carotene all downregulated the expression of TLR4, indicating that they attenuated the ability of the P-LPS to trigger TLR4-mediated signaling.

Another notable change revealed by 16S sequencing was that while kale supplementation reduced Enterobacteriaceae, it increased the representation of genus *Turicibacter*, a member of Firmicutes, by over 3–7-fold. This finding was confirmed by qPCR using primers specific to *Turicibacter* and the species *Turicibacter sanguinis* (Figure 8). *Turicibacter* are Gram-positive, strict anaerobes associated with anti-obesity and anti-inflammation effects [34]. *Turicibacter* have been linked to the tryptophan metabolism pathway. Indeed, PICRUSt2 showed that this bacterial pathway was highly enhanced in mice supplemented with kale. Abundance of *Turicibacter* negatively correlated with the DAI score and colon length (R^2^ = −0.87). The tryptophan metabolism pathway negatively correlated with the DAI score (−0.68) and positively correlated with abundance of *Turicibacter* (R^2^ = 0.72). However, the metabolism of *Turicibacter* and its interaction with the host and how it may be protective in the intestine are unclear. *Turicibacter* express the protein CUW_0748, which has sequence and predicted homology to mammalian serotonin (5HT) membrane transporter (SERT), which is responsible for the reuptake of serotonin in several organs, including the gut. The dysregulation of serotonin synthesis, release, receptor expression and reuptake could potentially be involved in the development of IBD [35,36]. In vitro tests have shown that *Turicibacter sanguinis* takes up serotonin and moderates genes involved in steroid and lipid metabolism and homeostatic mechanisms [34]. *T. sanguinis* may alleviate colitis by acting as a SERT receptor to reuptake serotonin in the intestinal lumen. However, our study did not show any role that *Turicibacter* may play in moderating the LPS effects. This will be one of our next research questions to answer.

A limitation of our study is the fact that no method of separately quantifying the amounts of P-LPS and A-LPS exists. There is no way of determining how much of each is formed in the gut. We based the assumption on the proportions of *Bacteroidota* vs. *Proteobacteria* and how these changed with each treatment. In conclusion, we showed that including kale in the diet before administering DSS is a nutritional modulation that protects against the severity of the resultant inflammation and associated disease. Through changing the gut microbiota, kale attenuates P-LPS production and promotes A-LPS production. Through augmenting the gut barrier function, it attenuates translocation of P-LPS and downstream signaling through the TLR4 signaling cascade, thus eliminating the downstream severe cytokine response. Including kale in the diet successfully manipulates the complex interplay between diet, microbiota and the host and is a strategy for altering the physiological status of the host and protecting against disease.

## Figures and Tables

**Table 1:** Diet formulations.

Ingredients (g)	HF	HF with 4.5% Kale
Casein	200	196
L-cystine	3	3
Corn starch	72.8	60.7
Maltodextrin 10	100	100
Sucrose	172.8	172.8
Cellulose, BW200	50	32.2
Soybean oil	25	24.24
Lard	177.5	177.5
Mineral mix	10	10
Dicalcium phosphate	13	13
Calcium carbonate	5.5	5.5
Potassium citrate	16.5	16.5
Vitamin mix	10	10
Choline bitartrate	2	2
Kale dried powdered	0	39
**Total (g)**	**858.15**	**862.49**
**Kcal from Macronutrients**		
Protein	716	716
Carbohydrate	1422.4	1422.4
Fat	1822.5	1822.5
**Total Kcal**	**3960.9**	**3961**

**Table 2:** Experimental set up.

DIET Group (Weeks 1–3)	Definition	Drinking Water in Week 3
HF (control diet)	HF diet with 45% fat	Tap water
HFKV	HF diet with 45% fat supplemented with 4.5% kale	Tap water
HF-DSS	HF diet with 45% fat	3.0% DSS in tap water
HFKV-DSS	HF diet with 45% fat supplemented with 4.5% kale	3.0% DSS in tap water

**Table 3:** Primers used for qPCR analysis of colon tissue.

Gene	Forward	Reverse
TLR4	AGT GCC CCG CTT TCA CCT CT	TCC GGC TCT TGT GGA AGC CT
iNOS	CAC CTT GGA GTT CAC CCA GT	ACC ACT CGT ACT TGG GAT GC
TNF-a	TAC TGA ACT TCG GGG TGA TTG GTC C	CAG CCT TGT CCC TTG AAG AGA ACC
NFKb	GAG TTT GCG GAA GGA TGT CT	TGT CTG CCT CTC TCG TCT T
IL-1ß	CCA GCT TCA AAT CTC ACA GCA G	CCA GCT TCA AAT CTC ACA GCA G
IL-6	TCC AGT TGC CTT CTT GGG AC	GTA CTC CAG AAG ACC AGA GG
TJP1	GCC ACT ACA GTA TGA CCA TCC	AAT GAA TAA TAT CAG CAC CAT GCC
MUC 2	TCA AAG TGC TCT CCA AAC TCT C	CCT CTC AGA ATT CCA CAC TCT T
Claudin-1	GTT TGC AGA GAC CCC ATC AC	AGA AGC CAG GAT GAA ACC CA
Occludin	CTC CCA TCC GAG TTT CAG GT	GCT GTC GCC TAA GGA AAG AG
F4/80	GGA AGG AAA TGG AGA GAA AG	GAA GAT CTA CCC TGG TGA AT
TBP	CCA GAA CTG AAA ATC AAC GCA G	TGT ATC TAC CGT GAA TCT TGG C

## Data Availability

The data generated or analyzed during this study are included in this published article; the Supplementary Information files and sequencing raw datasets can be found in an online repository. The names of the repository and accession numbers can be found at https://www.ncbi.nlm.nih.gov/sra/PRJNA976322.

## Supplementary Materials

The following supporting information can be downloaded at: https://www.mdpi.com/article/10.3390/nu15143222/s1. This manuscript contains four supplementary figures which include summarized data on (i) food consumption and water intake by the mice, (ii) all the full, uncropped Western blot pictures, (iii) NGS data showing the rarefaction summary for all four treatment groups, (iv) linear discriminant analysis of bacterial gene pathways that accounted for the differences between the four treatment groups. Figure S1: No differences in Food consumption and water intake were observed. Food intake was determined by weighing, food input, left over and spillage. Leftover tap water and DSS water in the drinking bottles from each experimental group was determined daily over the 7 days. (**A**). Daily food intake by weight. (**B**). Daily calculated calorie intake. (**C**). Daily water consumption. No difference in food intake and daily water intake was observed among control and DSS intervention groups. Figure S2A: Kale supplementation increased the expression of tight junction proteins and reduced expression of F4/80. Protein expression of tight junction proteins was determined by western blotting of colon tissue. (**A**). Protein expression of Occludin (**B**). Protein expression of β-actin on the same membrane with Occludin. (**C**) Protein expression of Claudin (**D**). Protein expression of β-actin on the same membrane with Claudin. Figure S2B: Kale supplementation decreased the expression F4/80. (**A**) Protein expression of F4/80. (**B**). Protein expression of β-actin on the same membrane with F4/80. Figure S3: Effects of Kale supplementation on the gut microbiome structure and diversity. Bacterial DNA was extracted from colon contents and used to sequence the 16S gene. Fastq files were used for bioinformatics analysis in QIIME2 to determine diversity and taxa abundance. (**A**). The rarefaction curves for all groups reached a plateau indicating that the sequencing depth was sufficient to detect majority of ASVs in each sample and capture the microbial diversity Rarefaction curves indicate that DSS reduced richness (less ASVs) and supplementing the diet with Kale attenuated this. Even in mice not given DSS, kale supplementation enhanced richness (**B**,**C**). Pairwise tests for differentiation in centroid location, i.e., the mean position of all the samples within each group (Permanova) and differences in within group variation (dispersion). No significant differences were detected. Figure S4. LDA of Bacterial gene pathways in the different groups. PICRUSt2 predicted a total of 150 functional pathways by comparing against KEGG orthologs. The LefSe analysis at a 2.5 threshold level and at a p-value of 0.05 in a non-parametric Kruskal-Wallis rank sum test showed that the 42 pathways above accounted for discriminative features between the 4 groups
