# Supplementary material for: The Vegetable ‘Kale’ Protects against Dextran-Sulfate-Sodium-Induced Acute Inflammation through Moderating the Ratio of Proinflammatory and Anti-Inflammatory LPS-Producing Bacterial Taxa and Augmenting the Gut Barrier in C57BL6 Mice
Source: Nutrients. 2023 Jul 20;15(14):3222. doi: 10.3390/nu15143222 (PMC10383939; doi:10.3390/nu15143222)
Supplement: Supplementary file 1 [file nutrients-15-03222-s001.zip › nutrients-2490636-supplementary.pdf]

The vegetable 'Kale' protects against dextran sulfate sodium-induced acute inflammation through moderating the ratio of proinflammatory and anti-inflammatory LPS-producing bacterial taxa and augmenting the gut barrier in C57BL6 mice

Samnhita Raychaudhuri[1], Md Shahinozzaman[1], Ujjwol Subedi[1] , Si Fan[1], Opeyemi Ogedengbe[1] and , Diana N. Obanda[1]

[1]Department of Nutrition and Food Sciences, University of Maryland, College Park, MD, USA.

Correspondence: Diana Obanda. Skinner Building Room 0112, University of Maryland, College Park, MD. Email: dobanda@umd.edu

SUPPLEMENTARY FILES

SUPPLEMENTARY DATA FOR PUBLICATION

This manuscript contains four supplementary figures which include summarized data on (i) food consumption and water intake by the mice, (ii) all the full uncropped western blots pictures, (iii) NGS data showing rarefaction summary for all four treatment groups, (iv) Linear discriminant analysis of bacterial gene pathways that account for differences between the four treatment groups

SUPPLEMENTARY FIGURES

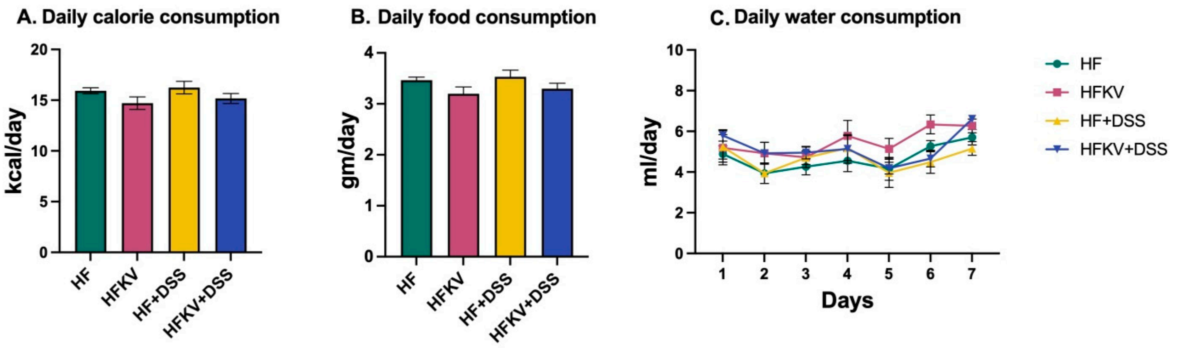

Supplementary Figure 1. No differences in Food consumption and water intake were observed.

Food intake was determined by weighing, food input, left over and spillage. Leftover tap water and DSS water in the drinking bottles from each experimental group was determined daily over the 7 days.

(A). Daily food intake by weight. **(B).** Daily calculated calorie intake. **(C).** Daily water consumption.

No difference in food intake and daily water intake was observed among control and DSS intervention groups.

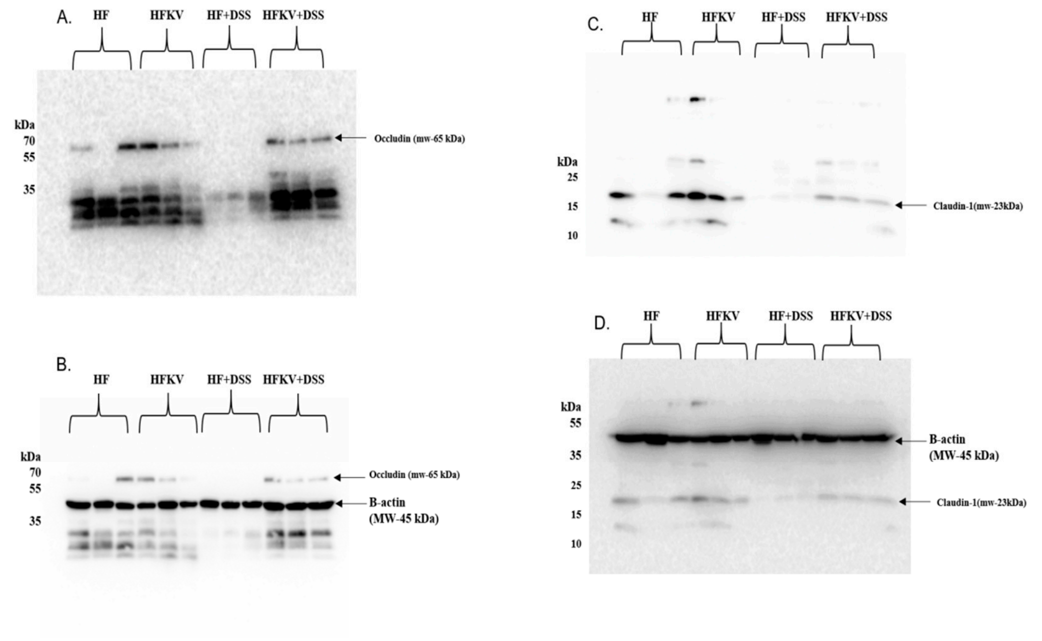

Supplementary Figure 2A. Kale supplementation increased the expression of tight junction proteins and reduced expression of F4/80

Protein expression of tight junction proteins was determined by western blotting of colon tissue. **(A)**. Protein expression of Occludin **(B)**. Protein expression of β-actin on the same membrane with Occludin.

(C) Protein expression of Claudin (**D).** Protein expression of β -actin on the same membrane with Claudin.

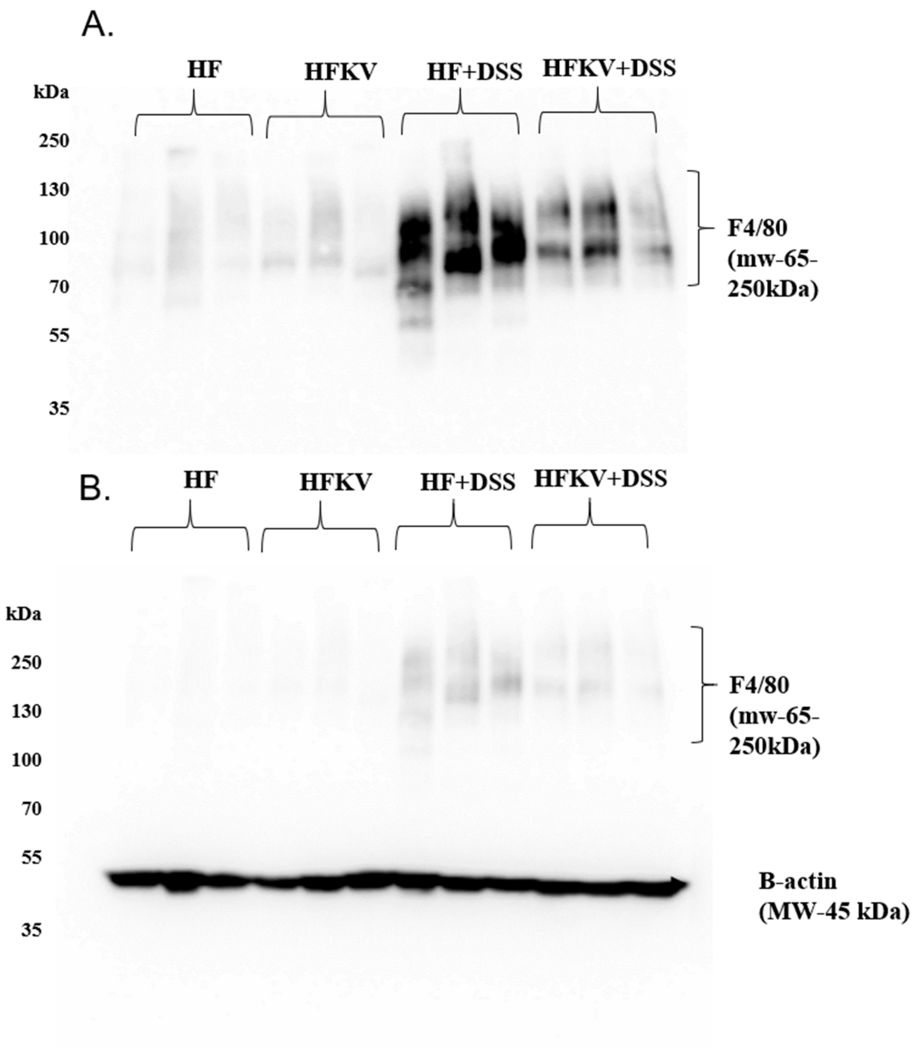

Supplementary Figure 2B. Kale supplementation decreased the expression F4/80

(A) Protein expression of F4/80. (**B).** Protein expression of β -actin on the same membrane with F4/80.

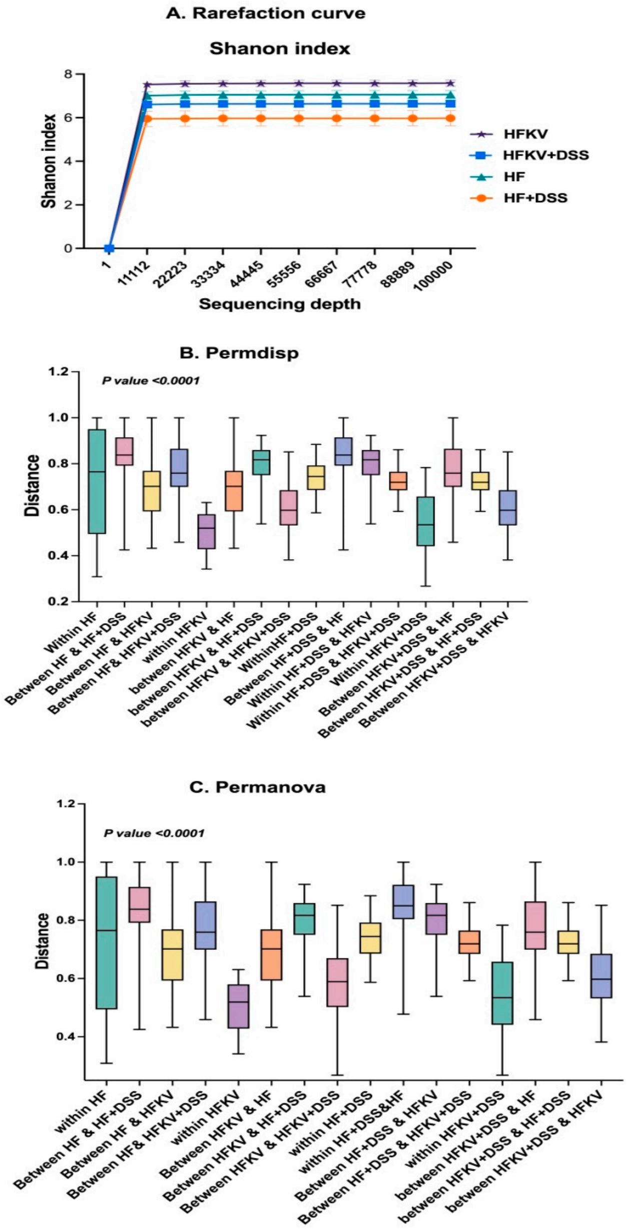

Supplementary Figure 3. Effects of Kale supplementation on the gut microbiome structure and diversity.

Bacterial DNA was extracted from colon contents and used to sequence the 16S gene. Fastq files were used for bioinformatics analysis in QIIME2 to determine diversity and taxa abundance.

(A). The rarefaction curves for all groups reached a plateau indicating that the sequencing depth was sufficient to detect majority of ASVs in each sample and capture the microbial diversity Rarefaction curves indicate that DSS reduced richness (less ASVs) and supplementing the diet with Kale attenuated this. Even in mice not given DSS, kale supplementation enhanced richness **(B and C).** Pairwise tests for differentiation in centroid location, i.e., the mean position of all the samples within each group (Permanova) and differences in within group variation (dispersion). No significant differences were detected.

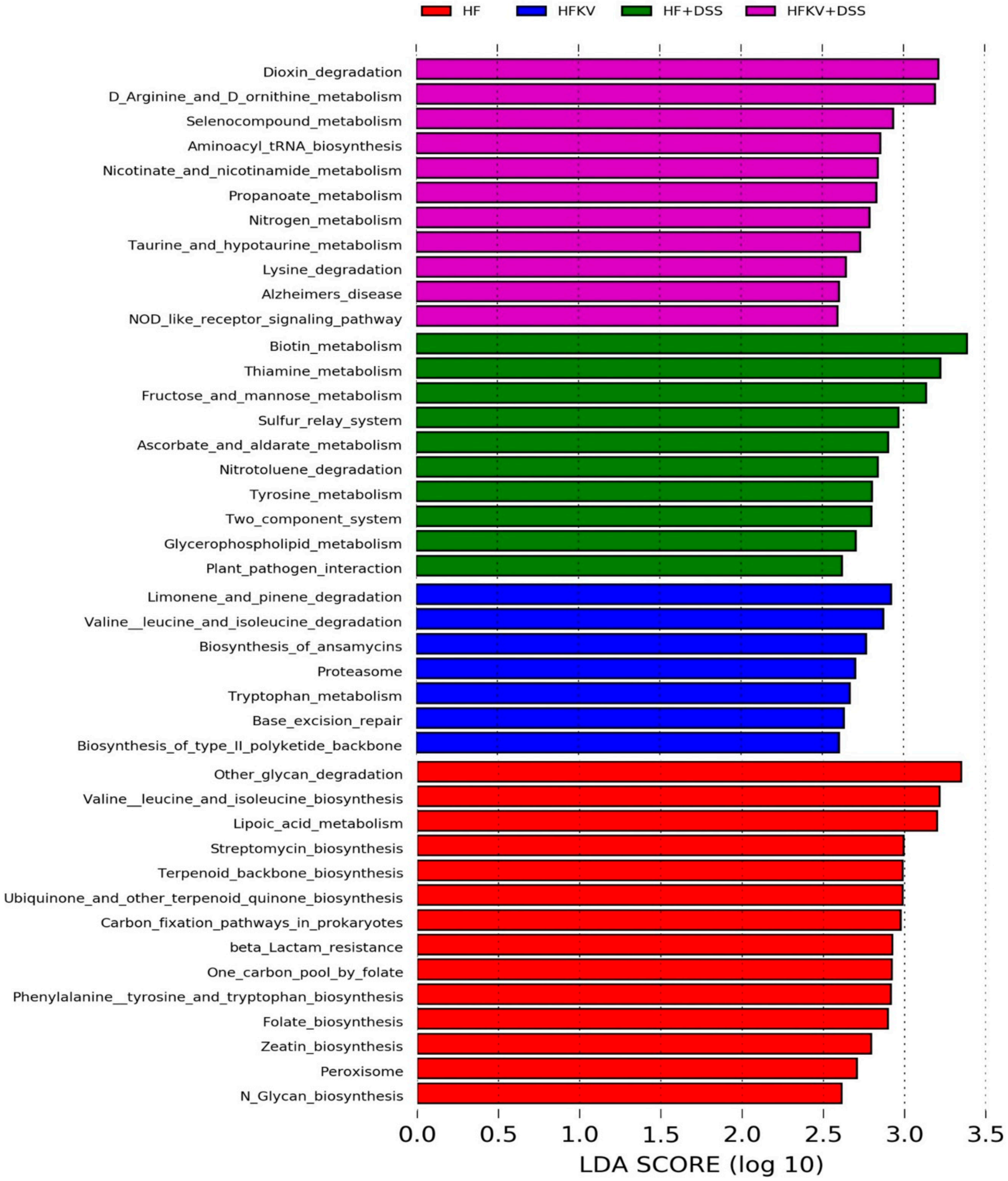

Supplementary Figure 4. LDA of Bacterial gene pathways in the different groups.

PICRUSt2 predicted a total of 150 functional pathways by comparing against KEGG orthologs. The LefSe analysis at a 2.5 threshold level and at a p-value of 0.05 in a non-parametric Kruskal-Wallis rank sum test showed that the 42 pathways above accounted for discriminative features between the 4 groups.
